# Comprehensive and Personalized Care of the Hemodialysis Patient in Tassin, France: A Model for the Patient-Centered Medical Home for Subspecialty Patients

**DOI:** 10.5402/2013/792732

**Published:** 2012-12-22

**Authors:** Eva Anvari, Hoda Mojazi Amiri, Patricia Aristimuno, Charles Chazot, Kenneth Nugent

**Affiliations:** ^1^Department of Internal Medicine, Texas Tech University Health Sciences Center, Lubbock, TX 79430, USA; ^2^Department of Internal Medicine, University of Arizona, 1501 N. Campbell, Tucson AZ 85721, USA; ^3^NephroCare Tassin-Charcot, 69110 Sainte Foy Les Lyon, France

## Abstract

The Centre de Rein Artificiel in Tassin, France, provides comprehensive care to patients with chronic renal disease similar to the model proposed for Patient Center Medical Homes; patients with end-stage renal disease in the Tassin Hemodialysis Center appear to have better outcomes than patients in the United States. These differences likely reflect this center's approach to patient-centered care, the use of longer dialysis times, and focused vascular access care. Longer dialysis times provide better clearance of small and middle toxic molecules, salt, and water; 85% of patients at the Tassin center have a normal blood pressure without the use of antihypertensive medications. The observed mortality rate in patients at the Tassin Center is approximately 50% of that predicted based on the United States Renal Data system standard mortality tables. Patient outcomes at the Tassin center suggest that longer dialysis times and the use of multidiscipline teams led by nephrologists directing all health care needs probably explain the outcomes in these patients. These approaches can be imported into the U.S healthcare system and form the framework for patient-centered medical practice for ESRD patients.

## 1. Introduction

 End-stage renal disease (ESRD) continues to increase in prevalence and incidence in developed countries. This has challenged health care systems to distribute resources in ways that control expenditures and achieve the best possible outcomes. The International Study of Health Care Organization and Financing (ISHCOF) compared 12 developed countries with different health care economic policies and assessed incentives, benefits, and outcomes. The United States had the second highest death rate per year among patients on hemodialysis (HD) in this study, even though it had the highest annual expenditure per ESRD patient and per capita among those 12 countries [1]. In addition, there is significant dissatisfaction among both patients and physicians with the current health care system in this country [2, 3]. In the 2011 Commonwealth Fund Survey, more than 7 out of 10 adults in the United States felt the need for major changes in the health care system [3]. They listed difficult care access, poor care coordination, excessive costs, and the administrative hassle of health insurance as their main reasons and favored more patient-centered policies [3].

 Because of the increasing need for change, the American College of Physicians, the American Academy of Family Physicians, the American Academy of Pediatrics, and the American Osteopathic Association have built a framework for patient-centered medical homes (PCMHs) as models for health care delivery [4]. The models depend on four essential features, including primary care, patient-centered care, new-model practice, and payment reform [5]. Primary care in the PCMH model provides comprehensive, first-contact, acute, chronic, and preventive care, delivered by a team of individuals led by the patient's personal physician. It also includes the essential primary care function of care coordination across multiple settings and clinicians. This model can be extended to selected subspecialty patients, including ESRD patients. Patient-centered care should meet the needs and preferences of patients, and the PCMH model expects active engagement of patients at all levels of care delivery, ranging from shared decision making to practice improvement. In addition, the PCMH model places the patient at the center of the health care system by expanding access and improving options for patient-clinician communication, such as use of the Internet for electronic “visits.” These PCMH “new” models should incorporate evidence-based processes of care, including population-based care management facilitated by patient registries, performance measurement and improvement, point-of-care decision support, and information technology. These efforts require extensive use of electronic clinical information technology. Payment reform includes a payment structure that combines fee-for-service, pay-for-performance, and a separate payment for care coordination and integration. Payment should compensate for care coordination, care management, and medical consultation outside the traditional face-to-face visit and recognize case-mix differences and technology costs. The American Society of Nephrology (ASN) appointed a taskforce to assess how nephrologists would interact with PCMH [6]. They recommended that the ASN endorses testing the PCMH concept and that nephrologists help create effective PCMHs. However, ideas about PCMH are still developing, and concerns about implementation and uncertainty about outcomes will likely slow this process [6]. 

 The Centre de Rein Artificiel in Tassin, France, provides one possible model for PCMH for patients with chronic renal disease and ESRD. This center is completely dedicated to kidney disease patients and serves as a “medical home” for them, meaning that it serves as the regular place of care for the patients, and the doctors and staff of this center often coordinate care received from other sources of care. It consists of a hospital, an operating room, a radiology suite, an outpatient clinic, four HD rooms, and a next door Chateau where patients do self-dialysis overnight. In this paper we consider the use of PCMH in patients with ESRD and discuss the relevance of the Tassin approach to this model [7–11].

## 2. Principal Dialysis Management Concerns: Dialysis Time and Vascular Access 

Longer dialysis times and better vascular access management improve outcomes in ESRD patients. In addition, adherence to clinical practice guidelines has improved survival and reduced hospitalizations and the use of resources [7–11]. In a recent prospective observational study, Lacson et al. tried to determine whether incremental achievement of up to eight quality of care indicators (goals) was associated with improvement in facility-specific mortality and hospitalization rates. These authors analyzed the results from 1085 dialysis centers in Fresenius Medical Care, North America (Waltham, MA) (FMCNA) of during the calendar year, 2006. They found that only 8% of the facilities met more than five quality goals and that the more goals achieved the lower the mortality and hospitalization rates [7]. The information from the FMCNA database suggests that three quality goals are strongly associated with decreased hospitalization and mortality. These include a *Kt*/*V* goal of 1.2 (dialyzer clearance of urea multiplied by time divided by the urea volume of distribution, an indicator of dialysis adequacy), consistent use of fistulas (fewer access complications), and good nutrition (albumin >3.8 g/dL) with decreased adverse events related to malnutrition. These factors represent essential considerations for this paper. 

In 1975 it was universally accepted that the gold standard for HD sessions was eight-hour sessions three times a week [12, 13]. As the demand for HD increased, as dialyzer technology improved, and as the concept of *Kt*/*V* evolved, the length of dialysis sessions was reduced [14]. However, despite these changes in standards, the center in Tassin continues to manage most of its HD patients with long HD sessions. This center adjusts each dialysis session according to the individual patient's clinical status and preferences. The length of the HD treatment typically ranges from four to eight hours and depends on the blood pressure, the nutritional status, the hemoglobin, and electrolyte abnormalities. The session time can vary throughout the patient's life and can change according to the patient's current clinical status. However, in the end, most patients (60%) are dialyzed for five to eight hours three times per week. This treatment provides a higher dose of HD which is more efficient in removing small and middle molecules, improves nutritional status, provides better control of anemia and hyperphosphatemia, and facilitates asymptomatic fluid removal by ultrafiltration [15]. Short HD sessions require increased ultrafiltration rates, and this leads to several adverse reactions, including more hypotensive episodes and symptoms, such as cramps, during the HD session [16, 17]. Lacson reported that the mean *Kt*/*V* for 84,989 patients in 1085 FMCNA facilities in the US in 2006 was 1.46 and that 45% of facilities met the quality goal of a *Kt*/*V* of 1.2 in 90% or more of patients. The *Kt*/*V* for urea clearance may oversimplify the analysis of dialysis efficacy because it neglects other major issues for patient outcomes such as phosphate clearance or extracellular fluid balance for which extended dialysis is very efficient.

 Vascular access-related complications account for most hospitalizations in patients undergoing HD, and access thrombosis or dysfunction can contribute to missed dialysis and consequently leads to higher morbidity and mortality [18, 19]. Frequent surveillance of fistula and grafts for stenosis improves patency rates and decreases the incidence of thrombosis. The Kidney Disease Outcomes Quality Initiative (KDOQI) guidelines recommend access surveillance with physical examination, direct flow measurements during the HD session, and duplex ultrasound. In Tassin, the nephrologists follow these recommendations closely, and every fistula is evaluated by a physician before HD sessions and during clinic visits. The patients with access problems are immediately referred to the radiologist for further evaluation and treatment. Angioplasty is done by the interventional radiologist if stenosis is detected. High output fistulas and aneurysms, detected by duplex ultrasound, are repaired by the nephrologists. Surveillance of the fistulas is also done by ultrasound annually even if no abnormalities are suspected. Compared to most institutions where the ultrasound needs to be ordered and interpreted before consultation with an interventional radiologist or vascular surgeon, at the Tassin center there is no delay in evaluation and treatment, and urgent problems are corrected within 48 hours. A multidisciplinary weekly meeting is dedicated to blood access complications. The quality goals for HD at FMCNA include both fistula placement in more than 50% of patients and HD catheters in less than 8%. Lacson reported that 47% of patients had fistulas and 22% of patients had HD catheters in 2006. More than 80% of patients at the Tassin center have fistulas.

## 3. Subspecialty Care Focus: Dry Weight and Blood Pressure 

 Intermediate outcomes in ESRD patients on dialysis include dry weight maintenance and blood pressure (BP) control. Dry weight is the body weight at the end of dialysis at which the patient can remain normotensive without antihypertensive medication despite fluid accumulation until the next dialysis [20]. One of the goals at the Tassin center is to have patients reach dry weight, and in the majority of the patients, this is achieved with a normal BP without the need for BP medications. Several factors are taken into consideration when assessing volume status, including blood pressure, physical exam findings of fluid overload, and trends in laboratory values (hematocrit and brain natriuretic peptide) and symptoms. During dialysis rounds, changes are made in the ultrafiltration and dry weight, and this may require an increase in the length of the hemodialysis sessions. There is always a discussion with the patient when changes need to be made, and this provides ongoing education to patients and families about dialysis goals. In addition, these changes require frequent clinical interactions with patients, and this likely improves the clinical decision making. The observational study by Lacson did not report either dry weight or BP control results, but the KDOQI clinical practice guidelines do include control of volume and blood pressure.

 With the reduction of HD time and changes in dialysate sodium concentrations, hypertension has been more difficult to control in dialysis patients. A high BP correlates with higher cardiovascular mortality and lower survival. In the Tassin center, a normal BP is achieved without medications in 85% of their patients [15]. Fluid is removed to decrease BP. The goal is to discontinue all blood pressure medications, which are removed one by one as the dry weight goal is reached. If BP remains elevated, more fluid is removed. The composition of the dialysis bath usually includes sodium concentrations of 138 mmol; this reduces interdialytic fluid intake since the patients tend to be less thirsty. Increased dialysis sodium concentrations which were introduced to minimize symptoms actually increase fatigue, leg cramps, and thirst; these symptoms lead to more water ingestion, increased extracellular volume, increased BP, and eventually left ventricular hypertrophy and decreased arterial compliance [21]. Another frequently used medication in HD patients associated with hypertension is erythropoietin (EPO) [15, 22]. Patients in Tassin have better controlled anemia and require less EPO, and this contributes to better BP control. The quality indicators for FMCNA HD centers do not include BP goals. 

## 4. Process Indicators: Phosphorus, Potassium, Hemoglobin, and Nutrition

Routine laboratory studies help monitor dialysis efficiency. High serum phosphate levels correlate with higher cardiovascular mortality and can be managed with longer dialysis sessions [23, 24]. In the Tassin center, only 35% of the patients on long HD use phosphate binders. The quality indicator for phosphorous levels is to maintain the phosphorous level below 5.5 mg/dL in 60% or more of patients. Lacson reported that 36% of the FMCNA centers met this goal. At the Tassin center, the average predialysis phosphate level is 4 mg/dL. Drastic fluctuations in serum potassium levels are associated with cardiac morbidity. To prevent this, potassium concentrations in the dialysate remain the same most of the time, and a few patients take sodium polystyrene sulfonate on a daily basis. 

Better control of anemia is another benefit of long HD. It has been reported that patients who meet the KDOQI goals for anemia have a lower mortality risk [25]. In the Tassin center, only 75% of the patients are on EPO with an average dose that is 50% of the dose used in the US [22]. The quality goal in FMCNA centers is a hemoglobin level greater than 11 g/dL in 80% or more of patients. Lacson et al. reported that this goal was met in 72% of centers. The average predialysis hematocrit in Tassin patients is 34.2%.

Protein malnutrition is very common in patients undergoing HD with a prevalence ranging from 18% to 70% [26–28]. Patients who lose weight during their first year of HD have a higher mortality rate than patients who have a stable or increased weight. The Hemo study demonstrated that there is a progressive decline of nutritional markers, including weight and albumin, in HD patients; however, a five-year follow-up study on Tassin patients with long HD demonstrated nutritional stability [29]. The long HD treatments allow for fewer dietary restrictions and more protein and calorie intake. The increase in lean body mass can be demonstrated by increased hematocrits, urea levels, and creatinine levels without changes in BP [12].

Therefore, the nutritional status is an important part of the overall management in the HD patients in Tassin. All patients are evaluated and followed by a dietitian. If malnutrition is diagnosed, the HD session is often prolonged. Other interventions implemented are similar to those followed in the US, including oral supplements, enteral, and parenteral feeding. The quality indicator for FMCNA dialysis centers is an albumin level ≥3.8 gm/dL in 65% of patients. Lacson reported that 9% of centers met this goal. The average protein intake in patients at the Tassin center is 1.2 gm per kg body weight per day.

## 5. Comorbidity

Dialysis management clearly influences outcomes. However, ESRD patients usually die from infections, cardiovascular disorders, and malignancy. Consequently, better outcomes will depend on attention to these disorders, and the PCMH could systematically organize this care.

## 6. Outcomes: Mortality 

 Several papers have reported higher mortality rates in patients with ESRD in the United States than in other countries. Some possible reasons include vascular access malfunction, following small solute clearance as the main measurement of dialysis adequacy, and inadequate nutrition [26, 30]. Using the standardized mortality ratio and the United States Renal Data system standard mortality table, the average observed mortality in the Tassin center is about 50% of the expected value for similar patients [23]. Innes et al. reported a similar result when patients at Tassin on long dialysis sessions were compared to patients in Nottingham, England, on conventional dialysis [31]. Patients in the Tassin center have the same demographics and the same causes of renal failure as other dialysis cohorts and have frequents histories of prior cardiovascular events. These patients also have better outcomes than other dialysis patients in France [12]. Lacson et al. reported lower mortality in a multicenter trial in the US which compared long dialysis sessions (7.9 hours) with conventional dialysis (3.7); this outcome clearly supports the thesis that longer dialysis times matter and can be achieved in the US [7]. 

## 7. Relevance to the PCMH

The Tassin center provides long HD sessions and comprehensive and individualized patient care. Patients are seen by a nephrologist during every dialysis session and by a radiologist, a dietitian, a social worker, and a psychologist if needed. These patients have lower mortality rates and fewer complications. Laurent has suggested that the combination of long HD sessions and more frequent evaluations by the nephrologists are the key factors in achieving better patient outcomes. We think that the approach to dialysis care and the outcomes at the Tassin center offer the following lessons. (1) Longer dialysis sessions provide better BP control and reduce mortality. (2) More frequent use of fistulas for dialysis is possible. (3) Proactive fistula care reduces complications. (4) Multidiscipline teams provide better outcomes and more patient education. The study reported by Lacson et al. demonstrates that process indicators can improve outcomes, but the number of indicators must be manageable. Recent studies by DaVita, Inc., and FMC demonstrate that comprehensive care programs in incident dialysis patients can reduce morbidity and mortality [32, 33].

 ESRD patients may represent the ideal patient population for the PCMH for subspecialty patients. (1) Patients on chronic dialysis have frequent contact with the health care system. Therefore, access is usually available, as centers are open six days per week. (2) Patients on chronic dialysis have complicated medical problems which often include hypertension, diabetes, coronary disease, anemia, and electrolyte disturbances. Complications related specifically to dialysis include vascular access failure and increased frequency of vascular access-related infections. Nephrologists clearly can manage these problems. Moving nephrologists' clinics into dialysis centers could significantly improve the quality of care through more frequent and timely care. (3) PCMH can institute protocols and guidelines relatively easily, can provide education to physicians, nurses, patients, and families, can track costs and outcomes, and can provide an excellent site for clinical trials. (4) PCMHs based in dialysis centers have the potential to improve the overall quality of care, provide a more specific focus on the management of chronic diseases, such as hypertension and diabetes, and provide rapid assessment of potential complications. (5) The Tassin experience indicates that this approach is possible and has desirable outcomes. (6) The organization of pilot projects involving nephrologists and dialysis centers has the potential to answer relevant questions in a relatively short period of time. The number of patients with chronic renal failure in the United States, the overall frequency of complications, and the availability of important and measurable outcomes make this an attainable goal. The organization, requirements for certification, and reimbursement for clinical care in PCMH will require significant input from all relevant parties. 

 Potential barriers include organizational structure, participation by nephrologists, and reimbursement. Existing dialysis centers provide a good start to the facilities needed for the PCMH. The nephrologists and especially the medical directors of dialysis centers must be committed to better outcomes [34]. These physicians must take the time to educate patients and nurses about the benefit of longer dialysis sessions. Nephrologists responsible for most of the ESRD patients care will likely have better outcomes than the nephrologists who only supervise dialysis care. Bundled reimbursement for both facility costs and professional costs coupled with outcome indicators will likely have powerful effects on organizational strategies [35]. 

## 8. Epilogue

 A recent report from the Dialysis Outcomes and Practice Patterns Study demonstrated that longer dialysis times reduced all cause and cardiovascular mortality and improved systolic blood pressure, hemoglobin levels, and potassium levels [36]. US patients had the shortest average dialysis time (214 ± 17 minutes); the longest times were in Australia/New Zealand (256 ± 23 minutes). In addition, recent changes in reimbursement in the US have resulted in lower hemoglobin levels and higher PTH levels [37]. This, of course, is not the desired outcome for improved care. Finally, DaVita (Denver, CO, a large dialysis chain) recently bought Health Care Partners (Torrance, CA, an owner of physician groups) for 4.42 billion dollars (Los Angeles Times, 5/22/2012). Dialysis services will change in the US. The main question is who will lead this process. More discussion about differences in patient populations will not advance patient care in the US or anywhere, and the main issue should be the outcomes [38]. 
